# Is there difference in chronic pain after Suture and Stapler fixation method of mesh in Ventral Hernia? Is stapler fixation method quicker? A randomized controlled trial

**DOI:** 10.12669/pjms.341.13904

**Published:** 2018

**Authors:** Noureen Shaukat, Farhat Jaleel, Masood Jawaid, Imrana Zulfiqar

**Affiliations:** 1 Dr. Noureen Shaukat, MBBS. Postgraduate Trainee, Department of Surgery, Dow University of Health Sciences, Karachi, Pakistan; 2 Dr. Farhat Jaleel, MBBS, FCPS. Associate Professor, Department of Surgery, Dow University of Health Sciences, Karachi, Pakistan; 3 Dr. Masood Jawaid, MBBS, MCPS, MRCS, FCPS, MHPE. Darul Sehat Hospital, Karachi, Pakistan; 4 Dr. Imrana Zulfiqar, MBBS. Assistant Professor, Department of Surgery, Dow University of Health Sciences, Karachi, Pakistan

**Keywords:** Chronic pain, Fixation method, Mesh repair, Suture, staple, Ventral hernia

## Abstract

**Background & Objective:**

Chronic pain occurs in 20–30% of patients after hernia surgery. As a consequence of this chronic pain, almost one third of patients have limitations in daily activities. Frequency and severity of this pain varies with different techniques of hernia repair. The objective of this study was to compare polypropylene suture and skin staples for securing mesh in uncomplicated ventral hernioplasty in terms of acute and chronic postoperative pain and to compare the time taken for mesh fixation between polypropylene sutures and skin stapler in ventral hernioplasty.

**Methods:**

This study was conducted in Surgery Department of Dow University Hospital, Dow University of Health Sciences, Ojha Campus and included 53 patients from Jan 2015 to Dec 2016, after taking informed consent. All patients were operated under general anesthesia by the same surgical team. Patients were randomized into two groups; in one group mesh fixed with 2/0 polypropylene suture while in other group mesh stapler was used. Time taken to apply mesh was noted in minutes from laying the mesh over anterior rectus sheath to completion of fixation by either method. The severity of post-operative pain was measured with VAS (1-10) after one week, one month and after one year after surgery. Data was analysed using SPSS version 17.

**Results:**

Patient characteristics and operative outcome were similar in the two groups and statistically non-significant in both. Early postoperative pain was more after suture fixation but it was not statistically significant. Mean ± SD pain score was after one week 3.47±2.7 after sutures while 2.91±1.88 after stapler. After four weeks, 0.40±0.49 after suture while 0.35±0.48 after stapler fixation. In both study groups 30–34% of the patients felt some pain in follow-up after one year. Severity of pain was 0.60±0.62 after suture while 1.65±1.94 after stapler fixation which is statistically significant as well (p<0.007). Mean operative time was 15.33±6.33 minutes for suture fixation while 1.56±0.41 minutes for fixation by staples, p-value < 0.001.

**Conclusion:**

The method of fixation does not appear to cause significant difference in early post-operative pain but chronic pain is more after stapler fixation of mesh. However, operative time was reduced significantly in staple fixation group as compared to suture fixation group

## INTRODUCTION

Ventral hernia is the bulging of part of the contents of the abdominal cavity through the musculoaponeurotic covering of the anterior abdominal wall. Ventral hernias include incisional hernia (15-20% of all abdominal wall hernias) and umbilical and epigastric hernias (10%) of hernias).^1^ Almost 150,000 ventral hernia repairs are being performed each year in USA.^1^ Primary suture repair was the standard treatment in past which carried high recurrence rates.^2^ Recurrence rates significantly decreased after the advent of mesh repair instead of primary suture repair.^2^ In ventral hernias, for defects larger than 2 cms in diameter, mesh repair is now the recommended method of treatment.^3^ The standard method of fixation of mesh on rectus sheath is with non-absorbable sutures.^4^ Reduction in operating time has been reported with skin staples instead of the standard technique of mesh fixation with sutures in inguinal hernia repair.^5^ Chronic pain after hernia repair is a matter of concern for the Surgeons. It is a complex problem and many risk factors are identified including the fixation of mesh by sutures.^6^,^7^ Method of mesh fixation is poorly studied and we cannot find randomized controlled trials comparing method of mesh fixation in open ventral hernia repair.^8^,^9^

The rationale of our study is to evaluate a method of mesh fixation for ventral hernia repair. Mesh fixation in ventral hernioplasty by skin staples is hypothesized to decrease chronic pain after hernia repair. Mesh fixation in ventral hernioplasty by skin staplers will take shorter time than by sutures. Less operative time will decrease infection rate. Less mesh handling in experimental group will again decrease operative time. More knots of suture material are expected to cause more acute and chronic pain in comparison to skin staples.

## METHODS

This randomized control study was conducted in Surgery Department of Dow University Hospital, Dow University of Health Sciences, Ojha Campus and included 53 patients from Jan 2015 to Dec 2016. The sample size was calculated by OpenEpi calculator. The mean pain score difference between two fixation techniques was taken as 0.7 (Sutures: mean ± SD = 3.2 ± 0.7 and Spiral tacks: mean ± SD = 2.5 ± 0.8).^8^ With 80% power, and 95% confidence level, the total sample size came out to be 38, at least 19 cases in each group.^10^ All patients with diagnosis of uncomplicated ventral hernia requiring meshplasty were included in the study. Patients with obstructed or strangulated hernia were excluded. Patients unwilling to participate or lost in one year follow-up were also excluded.

All patients after fulfilling the inclusion and exclusion criteria were verbally explained the procedure of mesh fixation in control and experimental groups. Written consent forms in English and Urdu were provided to the patients and collected after one week in the pre-operative outpatient clinic. Patients agreeing to be included in the study were randomized into two groups: group A in whom mesh was fixed with polypropylene suture and group B in which mesh was fixed with rotatable head skin stapler by Ethicon Johnson & Johnson.

All patients were operated under general anaesthesia. After dissection and opening the hernia sac, contents were reduced and repair with polypropylene suture no 1 was done. Tailored 15 × 15 cm mesh was used according to the size of hernia gap. Onlay meshplasty was done in group A with polypropylene suture no 2/0 and in group B with staples. Time taken to apply mesh was noted in minutes from laying the mesh over anterior rectus sheath to completion of fixation by either method. The severity of pain was measured with Visual Analogue Score (1 to 10) after one week, one month and one year of operation. All data was entered in Proforma.

### Statistical Analysis

All statistical analysis was performed using SPSS software version 17. Description of study variables were reported using descriptive statistics; mean and standard deviations were reported for continuous variables like age, BMI and operative time; and frequency and percentages were reported for categorical variables including sex, hernia site and size, hospital stay, and fixation techniques (outcome variable). Normality for continuous variables was assessed using Shapiro-Wilk Test (P-value >0.05). To assess the association between fixation techniques and all study variables Chi-square analyses for categorical variables and independent t-test for continuous variables were performed, for non-normal continuous variable Mann-Whitney test statistic was used. Association between Visual Analogue Score (VAS) and fixation techniques at different time periods was also assessed using Mann-Whitney nonparametric test, and all results were reported as p-values. Significant criteria was used as p-value <0.05.

## RESULTS

A total of 53 patients were included in the study that met inclusion and exclusion criteria, all selected individuals were randomly assigned to both fixation technique groups. After discharge, individuals were followed up at outpatient department at time period of one week, fourth weeks and one year.

Mean age of participants was (44.55±8.74), mean BMI (30.04±5.05), and females were dominant as compared to males (84.9% vs. 15.1%). Patients with suture fixation were 56.6% (n=30), and with staple fixation technique were 43.4% (n=23). Incisional and Paraumbilical hernia site were found more common.

Clinical and operative characteristics of patients according to fixation technique areshown in Table-I and Table-II. Comparisons between suture and staple techniques showed there were significant differences with BMI (p-value 0.034), hernia size (p-value 0.014) and operative time (p-value <0.001). Suture fixation (15.33±6.33) had higher operative time as compared to staple fixation (1.56±0.41). There was no significant difference between both technique groups in term of patient's age and hospital stay.

**Table-I:** Descriptive characteristics and fixation techniques (n= 53).

	Fixation Technique		

Individual characteristics	Suture (n = 30)	Staple (n = 23)	p-value
*Age in years*			
mean ± SD	46.47 ± 8.17	31.32 ± 4.49	0.068
*BMI*			
mean ± SD	42.04 ± 9.0	28.37 ± 5.35	0.034

	n (%)	n (%)	

*Sex*			
Male	8 (26.7%)	-	-
Female	22 (73.3%)	23 (100%)	

* p-value calculated using Independent t-test, SD: standard deviations, %: percentages.

**Table-II:** Clinical and operative characteristics with fixation techniques (n=53).

	Fixation Technique		

	Suture n (%)	Staple n (%)	p-value*
*Hernia site*			
Epigastric	4 (13.3%)	-	-
Incisional	12 (40.0%)	7 (30.4%)	
Incisional midline	-	2 (8.7%)	
Incisional Port site	-	2 (8.7%)	
Insicional (C-section)	4 (13.3%)	8 (34.8%)	
Paraumblical	10 (33.3%)	2 (8.7%)	
Umblical and epigastric	-	2 (8.7%)	
*Size of hernia (cm)*			
< 4 cm	4 (13.3%)	10 (43.5%)	0.014
≥ 4 cm	26 (86.7%)	13 (56.5%)	
*Hospital stay*			
2 days	16 (53.3%)	17 (73.9%)	0.126
3-4 days	14 (46.7%)	6 (26.1%)	
*Operative time (minutes)*			
mean±SD	15.33±6.33	1.56±0.41	< 0.001

* p-value calculated using Chi-square test, SD: standard deviations, %: percentages.

Postoperative pain scores in both fixation groups at different assessment periods were reported in Table-III. At first week, postoperative pain was found more severe after suture fixation as compare to staple fixation (mean score: 3.47 vs. 2.91), however it was not statistically significant. Similar assessment of pain scores was found at fourth week of follow-up. After one year, severity of postoperative pain was found significantly more among patients with staple fixation (1.65±1.94), as compare to pain scores with suture fixation (0.60±0.62), p-value=0.007.

**Table-III:** VAS pain scores at different time periods regarding to fixation techniques.

Postoperative time	Suture Fixation	Staple Fixation	p-value*
	mean ± SD	mean ± SD	
1 week	3.47 ± 2.70	2.91 ± 1.88	0.406
4 week	0.40 ± 0.49	0.35 ± 0.48	0.704
1 year	0.60 ± 0.62	1.65 ± 1.94	0.007

## DISCUSSION

Ventral hernia is a common condition accounting for about 150,000 hernia repairs being done in USA every year.^1^ Herniorrhaphy was initially considered the standard treatment and it carried very high recurrence rate. With the advent of different prosthetic meshes, recurrence rate has been considerably reduced, making mesh repair the recommended method of treatment. There are various methods for fixation of mesh and the choice is left on surgeon's preference as there is no established recommended method of mesh fixation. Our study has evaluated two methods of mesh fixation i.e. by polypropylene sutures or by staples with respect to operative time and chronic pain after open ventral hernia repair, as the quality of life has become an important matter of consideration for surgical procedures.

We found significant differences in mean BMI of both groups (mean BMI of suture group 42.04±9.0 versus that in staple group 28.37±5.35, p-value 0.034), however mean age was not different significantly in the two comparative groups (mean age of suture group 46.47±8.17 versus that in staple group 31.32±4.49 with p-value of 0.068). This was not consistent with the recent studies of Beldi G et al. done in 2011 and of Wessenaar E et al. done in 2010, as both studies have shown similar demographic parameters.^10^,^11^ Wessenaar E et al.^11^ also showed no statistically significant difference among the three study groups of mesh fixation techniques with respect to hernia size and hospital stay, however we have found a statistically significant difference between the two groups with respect to hernia size with p-value of 0.014 but no significant difference with respect to hospital stay (p-value 0.126).

In our study there was a significant difference in operative time between the two study groups with a p-value of <0.001, suture fixation (15.33±6.33) had a higher operative time as compared to staple fixation (1.56±0.41). This finding was consistent with three other studies of Dhillon RS et al., Khan AA et al. and Sheikh FA et al. done in 2008, 2014 and 2013 respectively.^5^,^12^,^13^

We have studied postoperative pain scores at one week, four weeks and one year in both groups. At first week pain was more severe after suture fixation than staple fixation but the difference was statistically nonsignificant. However chronic postoperative pain was more severe among patients of staple fixation group as compared to suture fixation with p-value of 0.007. These findings were not consistent with some studies as it has been reported that there is no significant difference in chronic pain after meshplasty between the study groups in the studies of Sheikh FA et al., Kitamura RK et al., Beldi G et al., Wessenaar E et al. and Colak E et al.^5^,^8^,^10^,^11^,^14^ However some studies have shown a statistically significant difference in early postoperative pain.^10^,^13^,^15^ However, some studies have demonstrated that there is no significant difference in early postoperative pain among the study groups.^8^,^11^,^14^

In a study done by Chatzimavroudis G et al. in 2017 it was concluded that none of the evaluated fixation method proved to be ideal.^16^

## CONCLUSION

The method of mesh fixation does not appear to cause any significant difference in early post-operative pain after meshplasty of open ventral hernia repair but chronic pain is more after stapler fixation of mesh. However operative time was reduced significantly in staple fixation group as compared to suture fixation group.

### Authors' contribution

**NS:** Did statistical analysis, manuscript writing, revising critically, editing and final approval of manuscript.

**FJ:** Conceived, designed, did data collection, editing, revising critically and final approval of manuscript.

**MJ and IZ:** Helps in data collection and final approval of the manuscript for publication.
